# Sodium intake and the risk of heart failure and hypertension: epidemiological and Mendelian randomization analysis

**DOI:** 10.3389/fnut.2023.1263554

**Published:** 2024-01-26

**Authors:** Miao Yuan, Dingyi Yan, Yu Wang, Mengyao Qi, Kexin Li, Zhi Lv, Dengfeng Gao, Ning Ning

**Affiliations:** ^1^Cardiology Diseases Department, The Second Affiliated Hospital of Xi'an Jiaotong University, Xi'an, China; ^2^Cardiology Diseases Department, Xi’an No. 3 Hospital, Xi'an, China; ^3^Cardiology Diseases Department, The Affiliated Hospital of Northwest University, Xi'an, China; ^4^Nuclear Medicine Department, Xi'an Jiaotong University Second Affiliated Hospital, Xi'an, China

**Keywords:** dietary salt, heart failure, hypertension, Mendelian randomization, NHANES

## Abstract

**Background:**

This study aimed to analysis the relationship between sodium intake and the risk of heart failure and hypertension through epidemiological studies and Mendelian randomization analysis.

**Methods and result:**

We initially conducted an analysis using data from the National Health and Nutrition Examination Survey (NHANES) database to examine the relationship between sodium intake and heart failure, hypertension, systolic blood pressure, and diastolic blood pressure. After adjusting for confounding factors, we found a non-linear association between sodium intake and heart failure (p nonlinear = 0.0448). Subsequently, we utilized Mendelian randomization (MR) analysis by utilizing urinary sodium as a proxy for sodium intake to investigate the relationships between sodium and heart failure, hypertension, systolic blood pressure, and diastolic blood pressure. The results indicated that with increasing urinary sodium, there is an increase in systolic and diastolic blood pressure, as well as an elevated risk of heart failure and hypertension.

**Conclusion:**

The evidence provided by this study suggests that higher sodium intake is associated with an increased risk of heart failure and hypertension. However, excessively low sodium intake may not necessarily be beneficial, as there may be maximum benefits at a sodium intake level of around 3,000 mg/d.

## 1 Introduction

Heart failure is a prevalent and severe cardiovascular disease that affects the quality of life and lifespan of millions of people globally (1, 2). Despite advances in medical management, heart failure remains a leading cause of incidence, hospitalization, and mortality (3, 4). One of the critical modifiable risk factors for heart failure is dietary sodium intake, and dietary sodium restriction has traditionally been a cornerstone of non-pharmacological therapy for heart failure (5–7). However, the relationship between dietary sodium intake and heart failure is complex and controversial, with many studies reporting conflicting finding (8, 9). While some studies suggest benefits (10, 11), others indicate better outcomes with sodium liberalization (12, 13). To better understand the relationship between dietary sodium intake and heart failure, we analyzed data from the National Health and Nutrition Examination Survey (NHANES) and conducted a Mendelian randomization analysis to investigate the causal relationship between urinary sodium excretion and heart failure. Mendelian randomization (MR) is an emerging epidemiological method that uses genetic variants as instrumental variables for exposure to estimate the causal effect on a specific outcome. This approach is less susceptible to confounding and reverse causality biases than observational studies (14, 15).

The aim of this study was to examine the relationship between dietary sodium intake and incident heart failure in NHANES patients and to test the causal relationship between urinary sodium excretion and heart failure using the Mendelian randomization method. We also discussed the clinical implications of our findings for the prevention and management of heart failure.

## 2 Materials and methods

### 2.1 Observational epidemiological analysis

The data analyzed in this study was obtained from NHANES, which is a program of the National Center for Health Statistics (NCHS) consisting of a series of continuous cross-sectional surveys. The database contains nutritional and health information for adults and children, including interviews and physical examinations. As NHANES is a publicly available database and all patients provided informed consent at the time of participation, ethical approval for this study was waived. The detailed NHANES study design and data are publicly available at https://www.cdc.gov/nchs/nhanes/. We downloaded and analyzed the data according to the tutorials of NHANES^1^ and survey content brochure.^2^ We included data from 2008 to 2018 and excluded individuals: (1) People younger than 20 years old, (2) Missing the data of sodium intake, (3) Missing data on diastolic and systolic or the pulse is irregular; (4) Missing data on heart failure or hypertension, (5) Missing the data on alcohol intake or smoking, and (6) Missing the data on marital status.

In this study, we extracted data on sodium intake from the dietary data section. In cases where an individual reported sodium intake on both the first and second day of the dietary interview, the average of the 2 days was calculated and taken as their sodium intake. However, if only the first day’s sodium intake data was available, it was used as their sodium intake value. We obtained the definitions of heart failure from the medical conditions mentioned in the questionnaire data, specifically MCQ160b (Ever told had congestive heart failure?). For hypertension, we relied on the response to question BPQ 120 (Ever told you had hypertension?) to determine its definition. A previous study demonstrated good correlation between self-reported cardiovascular disease and clinically confirmed (16, 17).

In this study, the SMQ120 (Smoked at least 100 cigarettes in life) was utilized to ascertain an individual’s smoking status based on the questionnaire data. The criterion used to differentiate between smokers and non-smokers was based on a lifetime consumption of fewer or more than 100 cigarettes. Similarly, the ALQ110 (Had at least 12 alcohol drinks/lifetime?) was employed to establish drinking behavior. Specifically, consuming more than 12 alcoholic drinks within the past year was classified as drinking, while a consumption of fewer than 12 drinks was classified as non-drinking.

Continine is a metabolite of nicotine, thus we use the level of continine in blood as a covariate to adjust for the effect of smoking on the risk of developing heart failure. The estimated glomerular filtration rate (eGFR) was calculated from serum creatinine levels using the following formula: eGFR = 175 x serum creatinine (mg/dl)^−1.154^ × age^−0.203^ × 1.212 (if black) × 0.742 (if female) (18). In addition to the above variables, we also incorporated age, sex, race, marital status, ratio of family income to poverty (PIR), body mass index (BMI), total cholesterol, serum sodium, education level as covariates in this analysis.

According to research, 90% of the world’s population has a sodium intake between 115–215 mmol/d (2,622–4,830 mg/d) (19). Considering that the Institute of Medicine (IOM) set the upper sodium intake (UL) at 2600 mg / day (20), we categorized the study population into four groups based on their sodium intake: low sodium diet (<2,600 mg/d), normal low sodium diet (2600–3800 mg/d), normal high sodium diet (3800–4800 mg/d), and high sodium diet (>4,800 mg/d). The distribution of data was assessed using the Kolmogorov test. Continuous variables and categorical variables were displayed using means or medians (interquartile range) and counts (frequencies), respectively, depending on the normality of the variable’s distribution. Baseline characteristics between groups were compared using analysis of variance (for normally distributed continuous data), chi-square test (for categorical variables), or Kruskal-Wallis H test (for non-normally distributed continuous data).

We used logistic regression to determine the relationship between sodium intake and outcome risk and adjusted for confounding factors. The 95% confidence interval (CI) of the odds ratio of the relationship between sodium intake and outcome was determined through multivariate adjustment. Three models were established: Model 1 was the crude model without confounder adjusted; Model 2 was adjusted for age, sex, and race; and Model 3 was further adjusted for age, sex, race, marital status, PIR, education level, alcohol intake, continine, total cholesterol, BMI, and eGFR. The low sodium diet group was used as the reference in all models. Finally, the restricted cubic spline (RCS) with four knots at the 5th, 35th, 65th, and 95th centiles was used to analyze the nonlinear relationship between sodium intake and heart failure (HF). We also used three models in RCS analysis to explore the nonlinear relationship between sodium intake and HF, hypertension, systolic blood pressure and diastolic blood pressure. In Model 1, no covariates were adjusted, in Model 2, sex, age, and race were adjusted, and in Model 3 age, sex, race, marital status, PIR, education level, alcohol intake, continine, total cholesterol, BMI, and eGFR were adjusted. Based on Model 3, we further constructed Model 4, 5 and 6 to adjust for systolic pressure, diastolic pressure, and hypertension respectively, as sodium intake may increase the risk of heart failure by affecting blood pressure. The likelihood ratio test was used to examine the nonlinearity.

Data analysis was performed using the Statistical Package for the Social Sciences (SPSS Statistics 26) and R (version 4.2.1). Statistical significance was determined by two-sided tests with a value of p threshold of less than 0.05.

### 2.2 MR analysis

**Figure 1:**
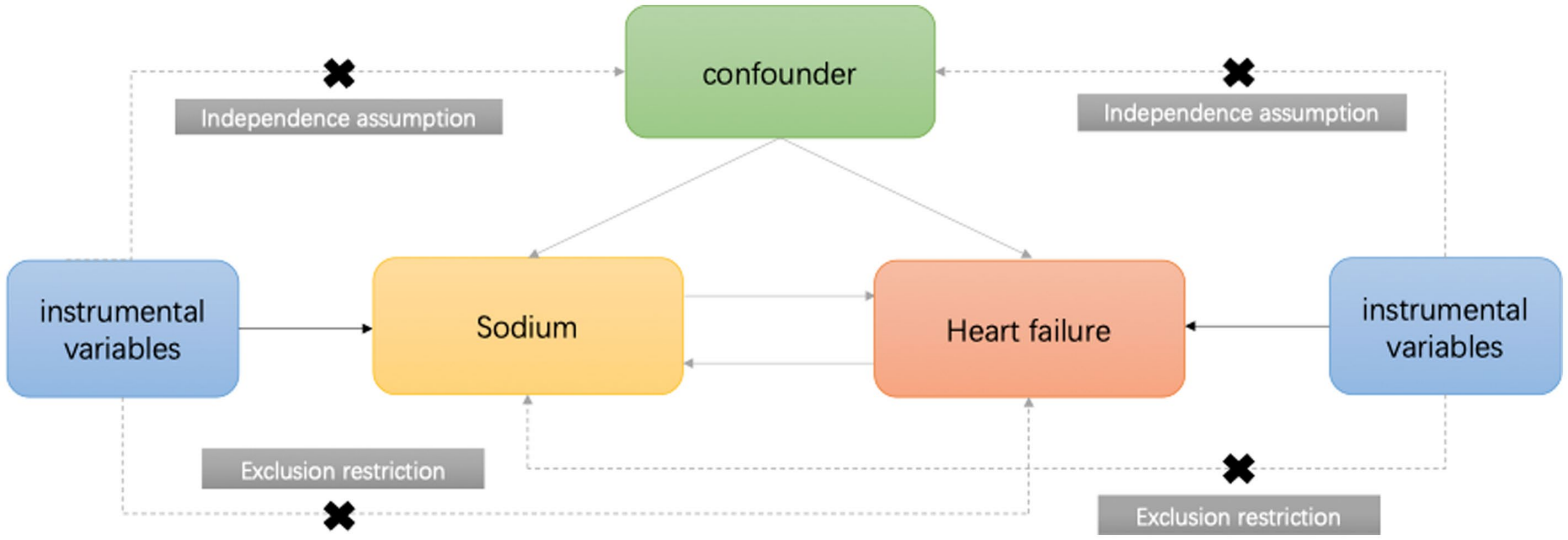
The key assumptions of MR analysis.

The summary data from the Genome Wide Association Study (GWAS) used in the MR analysis are publicly available and do not require an additional ethical statement. While there are no GWAS studies specifically related to dietary sodium, the estimation of sodium intake through urinary sodium measurement is a commonly used method. In this study, we utilized urinary sodium data from the UK Biobank,^3^ comprising 462,630 individuals predominantly of European ancestry, and employed two-sample Mendelian randomization (MR) to investigate the association between urinary sodium and HF, hypertension as well as blood pressure. We selected instructor variables (IVs) for MR analysis based on GWAS of the exposure data, and these IVs satisfied three key assumptions: (I) strong association with the exposure, (II) independence from potential environmental confounders, and (III) influence on the outcome risk only through the exposure variable (Figure 1). The outcome variable was derived from publicly available GWAS summary data, including heart failure, hypertension, systolic blood pressure, and diastolic blood pressure. Details of the dataset are provided in Supplementary Table S1.

We identified independent single nucleotide polymorphisms (SNPs) (*r*^2^ < 0.0001) associated with urinary sodium (*p* < 5×10^−8^) by analyzing GWAS summary data of urinary sodium from spot urine samples in the UK Biobank database. For each SNP, we calculated the *F*-value to assess the risk of weak instrument bias. An F-value greater than 10 indicates a low risk of weak instrument bias. The F-value for the instrumental variable was obtained by summing the *F*-values for each SNP. The calculation of F was based on the formula: *F* = *r*^2^ * (n−2) / (1−*r*^2^), where r^2^ was calculated using the following equation: *r*^2^ = 2 * effect allele frequency * (1−effect allele frequency) × *β*^2^ (*n* = sample size).

Given the potential for pleiotropy in genetic variation, we employed three methods to calculate MR for exposure and outcome after harmonizing effect alleles between exposure and outcome GWAS, including fixed-effects inverse-variance-weighted (IVW) method, MR-Egger, and weighted median. IVW provides a combined causal estimate for each SNP and is considered to have strong statistical power, so we used IVW as the primary analysis. IVW multiplicative random effects was used if there was heterogeneity between IVs and IVW fixed effects if there was no heterogeneity. MR-Egger allows for pleiotropy in all genetic variants, and the weighted median assumes that at least 50% of the information comes from valid instrumental variables. These two methods were used as supplements to IVW, as they can provide more robust estimates in a wider range of scenarios, albeit with lower efficiency (wider CI).

Horizontal pleiotropy occurs when genetic variants associated with the exposure of interest indirectly affect the outcome through pathways other than the assumed direct exposure. Therefore, sensitivity analysis was conducted. We used Cochran’s Q test to assess heterogeneity among different IVs in sensitivity analysis. When the *p* value of the Cochran’s Q test was less than 0.05, heterogeneity was detected. MR-Egger with a zero intercept (*p* > 0.05) was considered to have no pleiotropic bias. MR pleiotropy residual and MR-PRESSO methods were used to perform global heterogeneity tests and identify horizontal pleiotropy. Leave-one-out analysis was used to detect horizontal pleiotropy by sequentially removing SNP in the instrumental variable and calculating the impact of the remaining SNPs on the result. We also evaluated whether each SNP in the IVs had potential pleiotropy using the PhenoScanner website.^4^ SNPs with potential pleiotropy were removed, and IVW analysis was conducted again to avoid potential effects of pleiotropy on causal relationships.

In the MR analysis, we used the Bonferroni correction for *p*-values. As we had five outcome variables, the significance threshold was 0.0125 (0.05/4). *p* < 0.05, but >0.0125, was considered a potential association.

## 3 Results

### 3.1 Analysis of data from the NHANES database

We analyzed data from the NHANES database spanning from 2008 to 2018. Participants were selected based on predefined inclusion and exclusion criteria, as illustrated in Supplementary Figure S1, resulting in a total of 27,120 participants being included in the study. The median daily sodium intake was 3458.63 mg/d. Participants were divided into four groups based on their daily sodium intake, and the baseline characteristics of each group are presented in Table 1. The results demonstrate that individuals with lower sodium intake had a higher median age (*p* < 0.001) and a greater proportion of females (*p* < 0.001) compared to those with higher sodium intake. The group with higher sodium intake exhibited a higher percentage of individuals living alone (*p* < 0.001), and there were significant differences in sodium intake based on race (*p* < 0.001). Additionally, the group with lower sodium intake had significantly higher levels of continine and eGFR (*p* < 0.001), and there were differences in alcohol consumption and total cholesterol levels across the different sodium intake groups (*p* < 0.001). However, there were no significant differences in BMI or serum sodium levels across the groups (*p* = 0.811, *p* = 0.057).

**Table 1:** The baseline characteristics of different sodium intake group.

Total = 27,120	The group of sodium intake per day				*p* value
	<2,600 mg	2,600 ~ 3,800 mg	3,800 ~ 4,900 mg	>4,900 mg	
	*N* = 9,762	*N* = 8,074	*N* = 4,672	*N* = 4,612	
**Characteristics**					
*Age (years)*	54 (38–67)	52 (35–64)	47 (33–60)	42 (30–55)	<0.001
*Sex*					<0.001
Male	3,375 (34.6%)	3,748 (46.4%)	2,766 (59.2%)	3,395 (73.6%)	
Female	6,387 (65.4%)	4,326 (53.6%)	1906 (40.8%)	1,217 (26.4%)	
*Race*					<0.001
Mexican American	1,504 (15.4%)	1,181 (14.6%)	668 (14.3%)	759 (16.5%)	
Other Hispanic	1,158 (11.9%)	803 (9.9%)	450 (9.6%)	402 (8.7%)	
Non-Hispanic Blake	3,839 (39.3%)	3,513 (43.5%)	2069 (44.3%)	1922 (41.7%)	
Non-Hispanic White	2,308 (23.6%)	1,651 (20.4%)	957 (20.5%)	910(19.7%)	
Other Race	953 (9.8%)	926 (11.5%)	528 (11.3%)	619 (13.4%)	
*Education level*					
Less than 9th grade	1,257 (12.9%)	718 (8.9%)	351 (7.5%)	279(6.0%)	<0.001
9–11 grade	1,496 (15.3%)	1,046 (13.0%)	574 (12.3%)	637(13.8%)	
High school or equivalent	2,315 (23.7%)	1742 (21.5%)	1,082 (23.2%)	1,110 (24.1%)	
Some collage or AA degree	2,789 (28.6%)	2,468 (30.6%)	1,408 (30.1%)	1,476 (32.0%)	
Collage graduate or above	1905 (19.5%)	2,100 (26.0%)	1,257 (26.9%)	1,110 (24.1%)	
*Marital status*					<0.001
Living with partner	5,430 (55.6%)	4,861 (60.2%)	2,962 (63.4%)	2,834 (61.4%)	
Living alone	4,332 (44.4%)	3,213 (39.8%)	1710 (36.6%)	1778 (38.6%)	
*Smoke*					<0.001
Yes	4,132 (42.4%)	3,512 (43.5%)	2,143 (45.9%)	2,194 (47.6%)	
No	5,624 (57.6%)	4,562 (56.5%)	2,529 (54.1%)	2,418 (52.4%)	
*Alcohol*					<0.001
Yes	6,645 (68.1%)	6,043 (74.8%)	3,748 (80.2%)	3,838 (83.2%)	
No	3,117 (31.9%)	2031 (25.2%)	924 (19.8%)	774 (16.8%)	
*BMI*	28.12 (24.40–32.70)	28.2 (24.5–33.0)	28.20 (24.40–32.81)	28.33 (24.37–33.1)	0.057
*Continine (ng/ml)*	0.04 (0.011–15.325)	0.037 (0.011–8.103)	0.045 (0.011–30.68)	0.016 (0.066–65.38)	<0.001
*Total cholesterol (mmol/L)*	4.97 (4.27–5.72)	4.91 (4.24–5.66)	4.89 (4.19–5.64)	4.83 (4.16–5.56)	<0.001
*Glucose (mmol/L)*	5.22 (4.77–5.94)	5.16 (4.72–5.83)	5.16 (4.72–5.83)	5.16 (4.72–5.77)	<0.001
*Serum sodium (mmol/L)*	139.00 (138–141)	139 (138–141)	139 (138–141)	139 (138–141)	0.811
eGFR	93.01 (66.71–123.51)	98.70 (73.03–128.92)	105.91 (79.33–135.29)	115.64 (87.12–141.83)	<0.001
*Systolic blood pressure (mmHg)*	122.5 (111.5–136)	121 (111–133.5)	120.5 (111.5–131.5)	120.5 (112–130.5)	<0.001
*Diastolic blood pressure (mmHg)*	70 (62.5–77.5)	70.5 (63–78)	71.5 (64–78.5)	72 (64.5–79.5)	<0.001
*Hypertension*					<0.001
No	5,796 (59.4%)	5,210(64.5%)	3,099 (66.3%)	3,234 (70.1%)	
Yes	3,966 (40.6%)	2,864 (35.5%)	1,573 (33.7%)	1,378 (29.9%)	
*Heart failure*					<0.001
No	7,189 (96.1%)	12,453 (97.3%)	4,854 (97.7%)	1840 (98.1%)	
Yes	295 (3.9%)	340 (2.7%)	113 (2.3%)	36 (1.9%)	
*Coronary heart disease*					<0.001
No	7,143 (95.4%)	12,294 (96.1%)	4,807 (96.8%)	1833 (97.7%)	
Yes	341 (4.6%)	499 (3.9%)	160 (3.2%)	43 (2.3%)	

BMI, Body Mass Index; eGFR, estimated Glomerular Filtration Rate.

Table 2 compares the risk of developing HF and hypertension across the different sodium intake groups while including various covariates. In Model 1 which did not adjust for any covariates, the risk of HF and hypertension was lower in the normal and high sodium intake groups compared to the low sodium intake group (*p* < 0.001). In Model 2, after adjusting for sex, race, and age, the risk of HF was lower in the normal sodium intake group (normal low sodium diet and normal high sodium diet) compared to the low sodium intake group (*p* = 0.015, *p* = 0.003), while there was no significant difference in the risk of heart failure between the high and low sodium intake groups (*p* = 0.419). Different sodium intakes were associated with the risk of hypertension (*p* = 0.018), but a normal sodium diet (normal low sodium diet and normal high sodium diet) did not increase the risk of hypertension compared to a low sodium diet (*p* = 0.155, *p* = 0.373). The high sodium intake group did not exhibit a higher risk of hypertension compared to the low sodium intake group, but the value of p was borderline at 0.05 (*p* = 0.055). Model 3 included age, sex, race, marital status, PIR, education level, alcohol intake, continine, total cholesterol, BMI, and eGFR as covariates. The results indicated that the risk of HF was lower in the normal sodium intake group (normal low sodium diet and normal high sodium diet) compared to the low sodium intake group (*p* = 0.028, *p* = 0.002), and there was no significant difference in the risk of heart failure between the high and low sodium intake groups (*p* = 0.189). Furthermore, the risk of hypertension was lower in the normal (normal low sodium diet and normal high sodium diet) and high sodium intake groups compared to the low sodium intake group (*p* = 0.022, *p* = 0.985, *p* = 0.66).

**Table 2:** Multivariable-adjusted logistic regression analysis of the risk of heart failure (HF) and hypertension across various levels of sodium intake.

Variable	Model I		Model II		Model III	
Heart failure	OR (95% CIs)	*p* value	OR (95% CIs)	*p* value	OR (95% CIs)	*p* value
*Sodium intake*						
<2,600 mg	1		1		1	
2,600 ~ 3,800 mg	0.711 (0.600–0.842)	<0.001	0.804 (0.675–0.958)	0.015	0.819(0.685–0.979)	0.047
3,800 ~ 4,900 mg	0.538 (0.429–0.675)	<0.001	0.703 (0.556–0.890)	0.003	0.683(0.537–0.868)	0.010
>4,900 mg	0.545 (0.435–0.684)	<0.001	0.905 (0.710–1.153)	0.419	0.847(0.661–1.085)	0.416
*p* trend		<0.001		0.01		0.049
*Hypertension*						
*Sodium*						
<2,600 mg	1		1		1	
2,600 ~ 3,800 mg	0.803 (0.756–0.854)	<0.001	0.951 (0.888–1.019)	0.155	0.920 (0.857–0.988)	0.049
3,800 ~ 4,900 mg	0.742 (0.690–0.798)	<0.001	1.039 (0.956–1.129)	0.373	0.999 (0.916–1.089)	0.711
>4,900 mg	0.623 (−0.578–0.671)	<0.001	1.089 (0.998–1.189)	0.055	1.021 (0.932–1.118)	0.552
*p* trend		<0.001		0.018		0.047

Corrected covariates.

Model I without covariant.

Model II adjusted gender, age, race.

Model III adjusted age, gender, race, marital status, PIR, education level, alcohol intake, continine, total cholesterol, BMI, and eGFR.

PIR, ratio of family income to poverty; BMI, body mass index; eGFR, estimated glomerular filtration rate.

**Figure 2:**
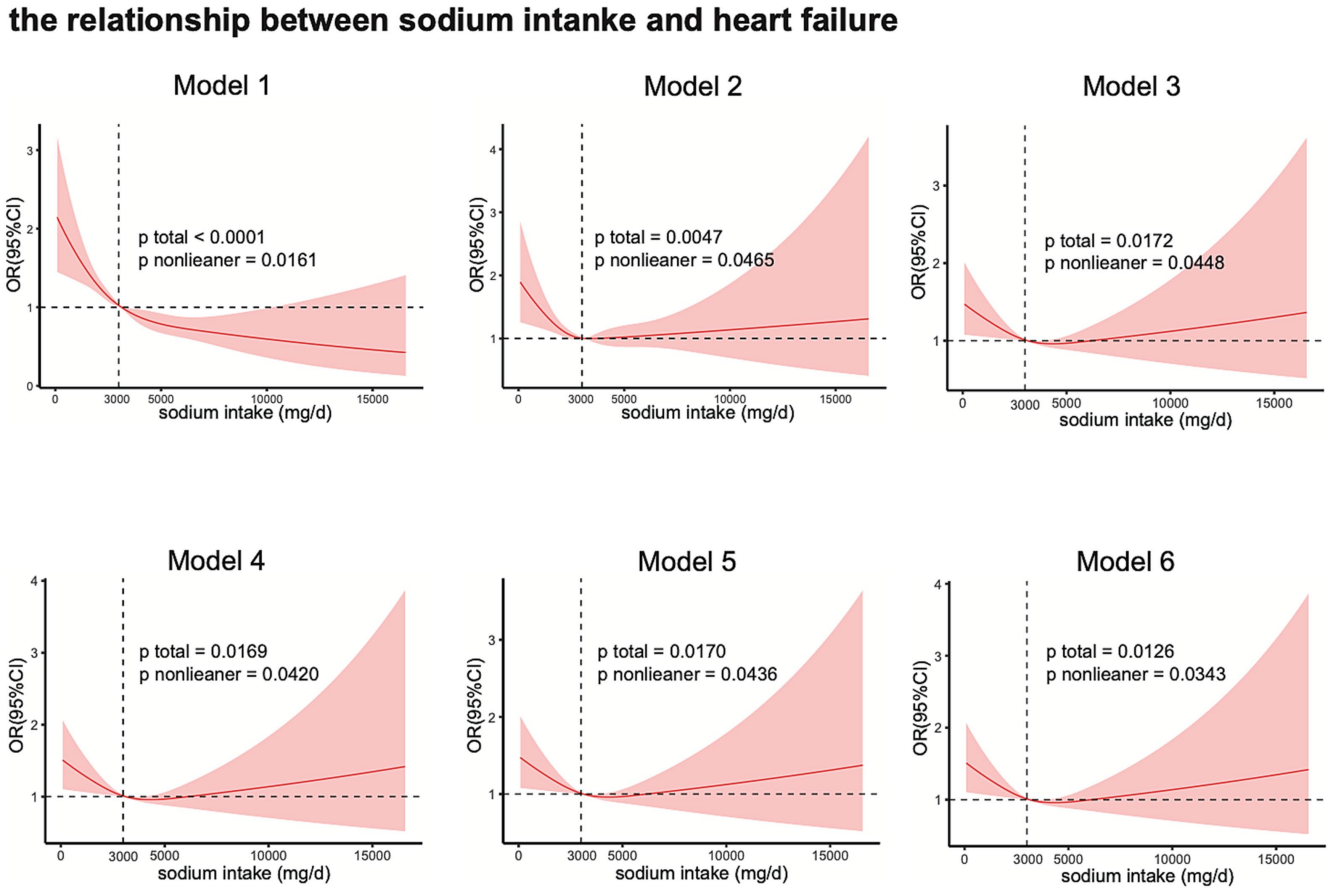
The relationship between sodium intake and the risk of heart failure.

As shown in Figure 2, the RCS analysis revealed an ‘L’-shaped non-linear relationship (*p* nonlinear = 0.0161) between HF and sodium intake without adjustment for covariates. In Model 2 and Model 3, a “U”-shaped relationship was observed (*p* nonlinear = 0.0465, p nonlinear = 0.0448). On the basis of Model 3, the Model4, Model5, Model6 adjusted for systolic pressure, diastolic pressure, and hypertension individually, the nonlinear relationship between sodium intake and the risk of heart failure remains (p nonlinear = 0.0420, p nonlinear = 0.0436, p nonlinear = 0.0343). As shown in Supplementary Figure S2 in Model 1 an ‘L’-shaped relationship was observed between sodium intake and hypertension (*p* nonlinear = 0.0018), but there was no non-linear relationship between sodium intake and hypertension in Model 2 and Model 3 (*p* nonlinear = 0.2508, *p* nonlinear = 0.2457). In Model 1 and Model 2 sodium intake had a non-linear relationship with SBP and DBP (*p* nonlinear for SBP < 0.0001, p nonlinear for SBP = 0.0020, p nonlinear for DBP = 0.0044, *p* nonlinear for DBP = 0.0403). As shown in Model 3, after adjusted for all covariates the nonlinear relationship between sodium intake and SBP disappears (*p* nonlinear for SBP = 0.0513) (*p* nonlinear for DBP = 0.5789).

### 3.2 MR analysis

We used 29 SNPs associated with urinary sodium as instrumental variables and their characteristics are presented in Supplementary Table S2. The *F* value of each SNP was greater than 10. We employed these instrumental variables to investigate the associations of urinary sodium with heart failure, hypertension, systolic blood pressure, and diastolic blood pressure. We found no significant SNP-outcome associations when we searched for the roles of these SNPs on Phenoscanner.

Table 3 shows that the Cochrane Q test detected heterogeneity for heart failure, hypertension, systolic blood pressure, and diastolic blood pressure (*p* < 0.05). To address this issue, we applied multiple random effects in the IVW analysis for these outcomes. Moreover, the intercept *p*-value was greater than 0.05 for all outcomes, indicating no evidence of horizontal pleiotropy. Notably, the MR-PRESSO global test revealed a potential pleiotropy for heart failure, hypertension, and diastolic blood pressure (*p* < 0.001).

**Table 3:** Results of potential pleiotropy and heterogeneity assessments.

outcome	Cochran’s Q statistic	*p*-value for Cochran’s Q	*p*-value for intercept	MR-PRESSO global test
HF	63.377	3.525E-05	0.8312526	<0.001
Hypertension	100.18118	2.40E-10	0.7518133	<0.001
Systolic	38.82936	0.03831589	0.4537263	0.046
Diastolic	391.9251	2.20E-66	0.4084242	<0.001

HF, heart failure.

**Figure 3:**
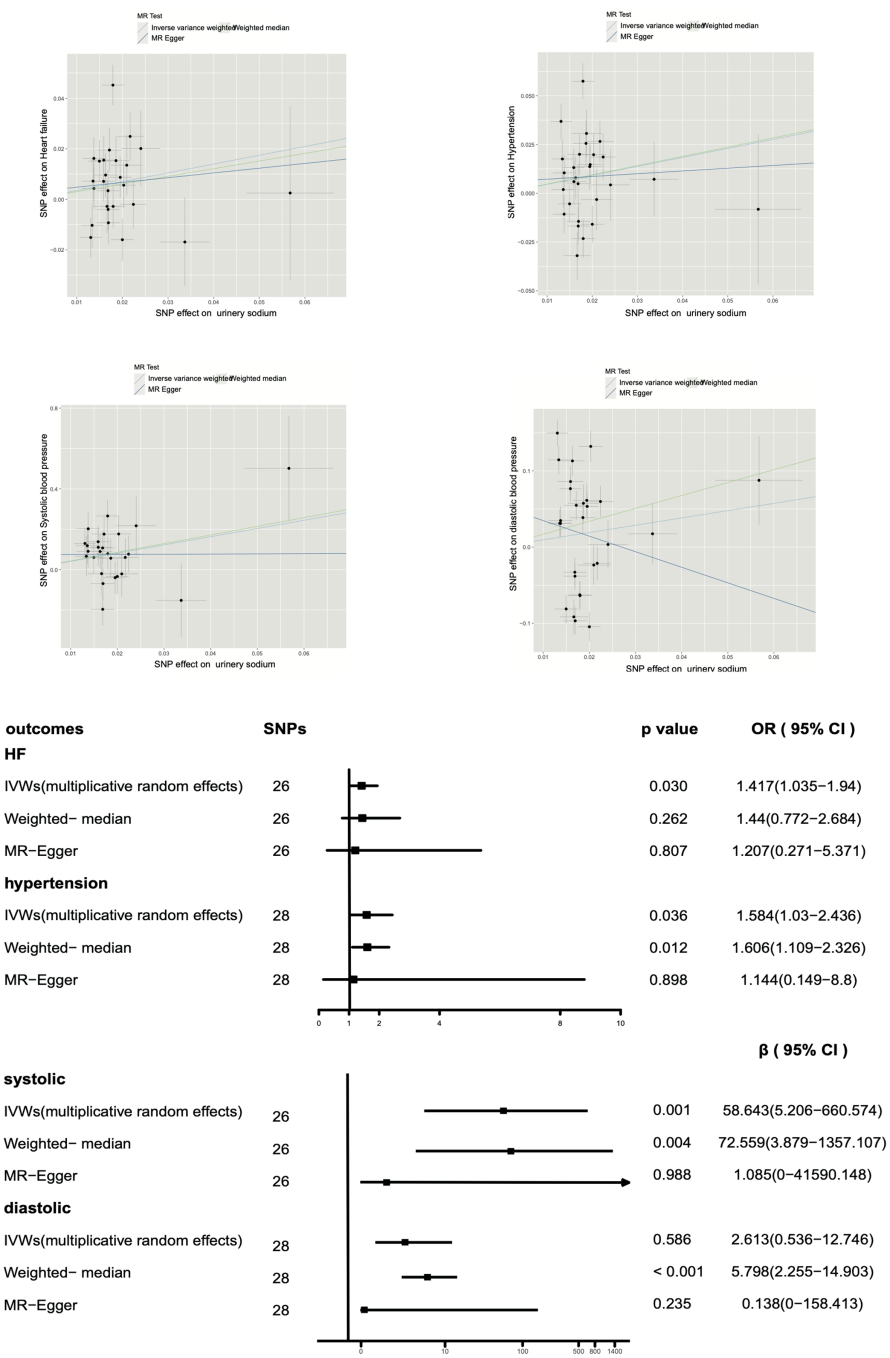
Impact of urinary sodium on heart failure risk and hypertension using Mendelian randomization.

Furthermore, as shown in Figure 3, our multiplicative random effects IVW estimates suggest a potential correlation between urinary sodium and heart failure (*p* = 0.030, OR 1.417, OR 95% CI 1.035–1.940). A similar result with broader CI was obtained through the weighted median (*p* = 0.262, OR 1.44, OR 95% CI 0.772–2.684) and MR-Egger test (*p* = 0.807, OR 1.207, OR 95% CI 0.71–5.371). The present study reveals a significant association between urine sodium and hypertension in both the inverse-variance weighted (IVW) test and weighted median analysis (IVW *p* = 0.036, OR 1.584, OR 95% CI 1.03–2.436; weighted median *p* = 0.012, OR 1.606, OR 95% CI 1.109–2.326). Results from MR-Egger analysis also indicated a similar association, but with wider CI (*p* = 0.898, OR 1.144, OR 95% CI 0.149–8.80).

Moreover, the leave-one-out and single SNP analyzes illustrated that the overall effect was not driven by any individual SNP between urinary sodium and HF, hypertension, systolic blood pressure or diastolic blood pressure (Supplementary Figure S3).

Moreover, a positive correlation was observed between urinary sodium and systolic blood pressure (IVW *p* = 0.00098), and a potential correlation was suggested by the weighted median analysis (*p* = 0.004), with similar results obtained from MR-Egger (*p* = 0.988). However, no correlation was found between urinary sodium and diastolic blood pressure in the IVW analysis (*p* = 0.586) or MR-Egger analysis (*p* = 0.235), and a positive correlation was suggested by the weighted median analysis (*p* < 0.001).

## 4 Discussion

This study used epidemiological analysis and two-sample MR analysis to investigate the causal relationship between sodium intake and the risk of HF and hypertension. We found that high sodium intake is associated with an increased risk of heart failure and hypertension, and that the relationship between high sodium intake and heart failure risk is nonlinear. This suggests that lower sodium intake does not necessarily confer greater benefits, but maintaining sodium intake within an appropriate range may yield greater benefits.

Many studies have explored the relationship between sodium and cardiovascular disease. In patients with type 2 diabetes, reduced 24-h urinary sodium excretion is paradoxically associated with increased all-cause and cardiovascular mortality (21). Both low and high sodium intake were associated with all-cause mortality and the duration of cardiovascular disease (22, 23). In most national and international guidelines recommended minimizing sodium intake (24–26), however, increasing evidence suggests that very low sodium intake does not necessarily reduce the incidence of cardiovascular disease (27) or heart failure (28–30). The benefits of restricting sodium intake may have been overestimated.

Research on the relationship between sodium intake and blood pressure is extensive and far-reaching. The linear relationship between sodium intake and blood pressure has been confirmed in multiple studies (31, 32), and our study reached a similar conclusion: after adjusting for confounding factors, there is a linear relationship between sodium intake and diastolic blood pressure and systolic blood pressure. When analyzing the relationship between sodium intake and blood pressure, we found that even with an increase of 100 mmol in sodium intake, the increase in blood pressure is limited (approximately 4 mmHg for systolic blood pressure and 2 mmHg for diastolic blood pressure). This limited effect can explain why we did not find a correlation between high sodium intake and the incidence of hypertension after adjusting for all confounding factors. This contradicts previous research results, and we believe it may be due to the fact that the population’s sodium intake is relatively concentrated. A smaller number of people have very high sodium intake, and the limited number of them having hypertension results in a wide 95% confidence interval for our odds ratio and non-significant results. Is sodium intake related to the risk of developing heart failure through its influence on blood pressure? We found that even after correcting for blood pressure, there still exists a non-linear relationship between sodium intake and the risk of heart failure. The impact of dietary sodium on the cardiovascular system may not solely be mediated by blood pressure. Previous studies have shown that high sodium intake can directly lead to ventricular remodeling (33, 34). We believe that sodium does not affect the pathogenesis of heart failure solely through its impact on blood pressure but rather through distinct mechanisms.

The relationship between sodium intake and heart failure is complex, as high salt intake exacerbates sodium and water retention, thereby aggravating heart failure symptoms and disease progression (35). Low salt intake is the main dietary strategy for treating heart failure. However, the benefit of very low sodium intake for heart failure is controversial. A study by Hummel et al. (36) of 443 heart failure patients with preserved systolic function showed that recommendations for salt restriction were associated with lower readmission and mortality rates within 30 days of discharge. In contrast, a recent randomized trial by Paterna et al. (37) showed that lower salt intake has adverse effects on the kidneys and neurohormones. How much should the salt intake of heart failure patients be reduced? Unfortunately, there is no clear evidence to answer this question.

Our study results indicate that even after adjusting for confounding factors, there is still a positive correlation between high sodium intake and the risk of heart failure. Restricted cubic spline analysis revealed that a lower sodium intake did not necessarily result in a lower risk of heart failure. The non-linear relationship between sodium intake and heart failure. When the sodium intake is less than 3,000 mg/d, we can observe that there is a negative correlation between sodium intake and heart failure with a 95% confidence interval that does not include 1. While a positive correlation between sodium intake and heart failure can be seen when the sodium intake is above 3,000 mg/d, the 95% confidence interval for the odds ratio is wide and even crosses 1. Therefore, we believe that higher sodium intake below 3,000 mg/d can actually decrease the incidence of heart failure. Unlike hypertension, the relationship between sodium intake and heart failure follows a U-shaped curve. Our study results suggest that the lowest risk of heart failure occurs at a sodium intake level of around 3,000 mg/d. This happens to be the average sodium intake for most people’s diets, so we believe that salt restriction may not necessarily lower the risk of heart failure.

To supplement and validate our cross-sectional investigation, we employed Mendelian randomization to yield congruent outcomes using diverse methodologies, thereby substantiating the reliability of our study findings. Mendelian randomization analysis confers several merits and carries profound implications for drawing deductions. One of the primary strengths lies in its capacity to furnish evidence for causal relationships. By employing genetic variants as instrumental variables, it surmounts confounding and reverse causality biases frequently encountered in observational studies. The utilization of genetic variants in Mendelian randomization introduces a facet of inherent randomization. As these variants are randomly assigned during meiosis, they remain unaffected by extraneous factors or confounding variables. This process emulates the random allocation of participants in a randomized controlled trial, fortifying Mendelian randomization studies against bias. This study benefits from the substantial sample size of GWAS data. Mendelian randomization analysis affirms that an escalation in urinary sodium corresponds to an elevation in both systolic and diastolic blood pressure. The higher urinary sodium engendering an augmented risk of hypertension and heart failure. These findings harmonize with the outcomes obtained from cross-sectional investigations.

Our study provides a new direction for the impact of sodium intake on cardiovascular disease. Lower sodium intake does not necessarily reduce the risk of disease occurrence, and maintaining sodium intake within a normal range may have greater benefits. This value is approximately 3,000 mg per day. A very low sodium intake, which means far below the normal sodium intake, may not necessarily bring benefits. Our research is based on analyzing the estimated sodium intake in the diet, which cannot accurately reflect the intake of sodium during evaluation. Therefore, further research is needed to determine whether a lower sodium intake is harmful or not. There is still much debate about sodium intake, and all of this remains inconclusive, indicating that large-scale studies may be necessary to obtain more valuable conclusions.

There are some limitations to our research. Instead of 24 h urinary excretion of sodium, NHANES obtained information about dietary sodium intake through a questionnaire survey, which may differ from individual’s actual daily sodium intake. In addition, Mendelian randomization was analyzed using urinary sodium, while cross-sectional studies were analyzed using dietary sodium. A recent study found that there may be differences in evaluating the relationship between sodium intake and disease through 24-h urine excretion, spot urine sodium (38). In addition, this study is a cross-sectional study, and there may be a causal reversal relationship. Although we attempted to avoid this limitation as much as possible through MR analysis, prospective randomized controlled studies are still necessary.

## Data availability statement

The data that support the findings of this study are available from the corresponding author upon reasonable request. The National Health and Nutrition Examination Survey (NHANES) database used in this study is publicly available and can be accessed at https://www.cdc.gov/nchs/nhanes/index.htm. In addition, the GWAS data used in this study are available from the following sources: exposure data (urinary sodium) can be downloaded from the UK Biobank (https://www.ukbiobank.ac.uk/), outcome data for heart failure can be downloaded from the Hermès Consortium (https://www.hermesconsortium.org/), blood pressure data can be downloaded fromIEU (https://gwas.mrcieu.ac.uk/files/ieu-b-4817/ieu-b-4817.vcf.gz), and systolic blood pressure data can be downloaded from the Within-Family GWAS Consortium (https://www.withinfamilyconsortium.com/home/).

## Author contributions

MY: Data curation, Formal analysis, Writing – original draft, Writing – review & editing. DY: Data curation, Formal analysis, Writing – original draft, Writing – review & editing. YW: Data curation, Formal analysis, Writing – review & editing. KL: Data curation, Writing – review & editing. ZL: Writing – review & editing, Data curation. DG: Funding acquisition, Project administration, Supervision, Writing – review & editing. NN: Project administration, Supervision, Writing – review & editing. MQ: Writing – original draft, Writing – review & editing.

## Footnotes

^1^https://wwwn.cdc.gov/nchs/nhanes/tutorials/default.aspx

^2^https://wwwn.cdc.gov/nchs/data/nhanes/survey_contents.pdf

^3^https://www.ukbiobank.ac.uk

^4^http://www.phenoscanner.medschl.cam.ac.uk
