# Supplementary figures and images for: Sodium intake and the risk of heart failure and hypertension: epidemiological and Mendelian randomization analysis
Source: Front Nutr. 2024 Jan 26;10:1263554. doi: 10.3389/fnut.2023.1263554 (PMC10853369; doi:10.3389/fnut.2023.1263554)

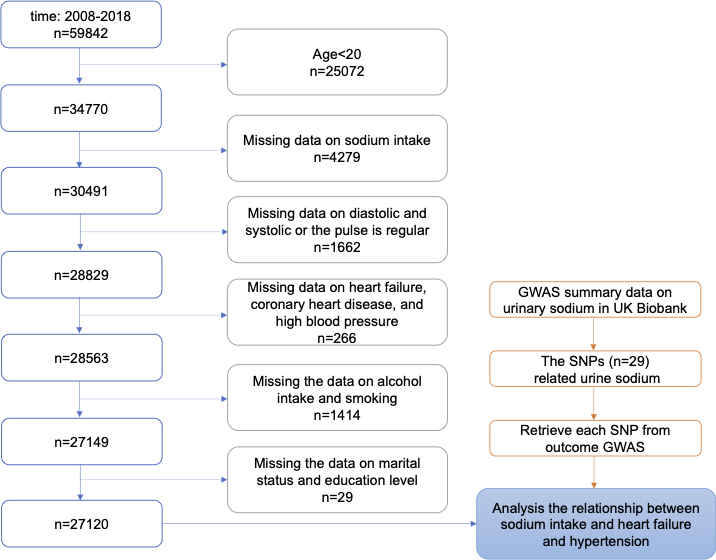

Supplement: Supplementary file 1 [file Image_1.TIFF]

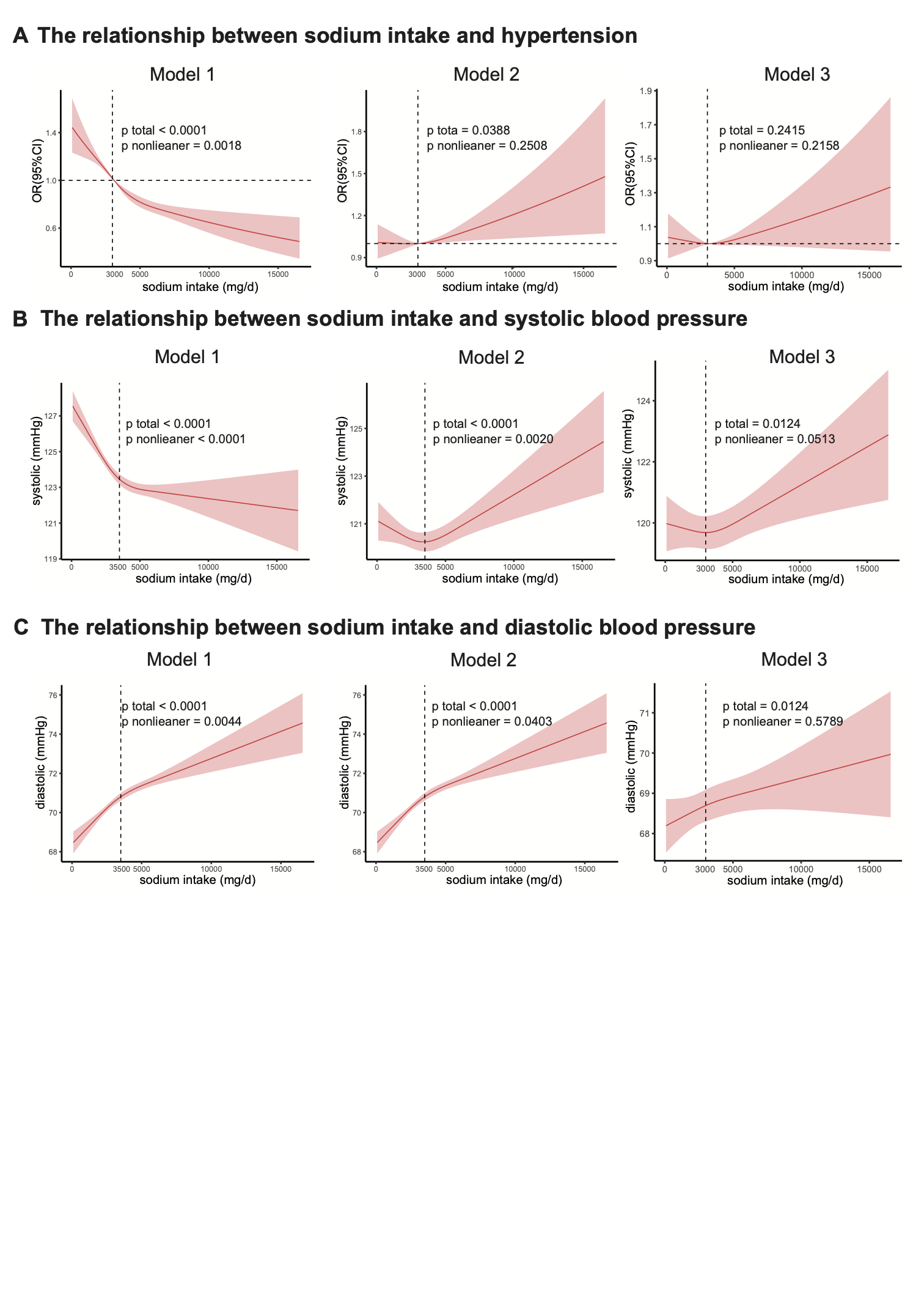

Supplement: Supplementary file 2 [file Image_2.TIFF]

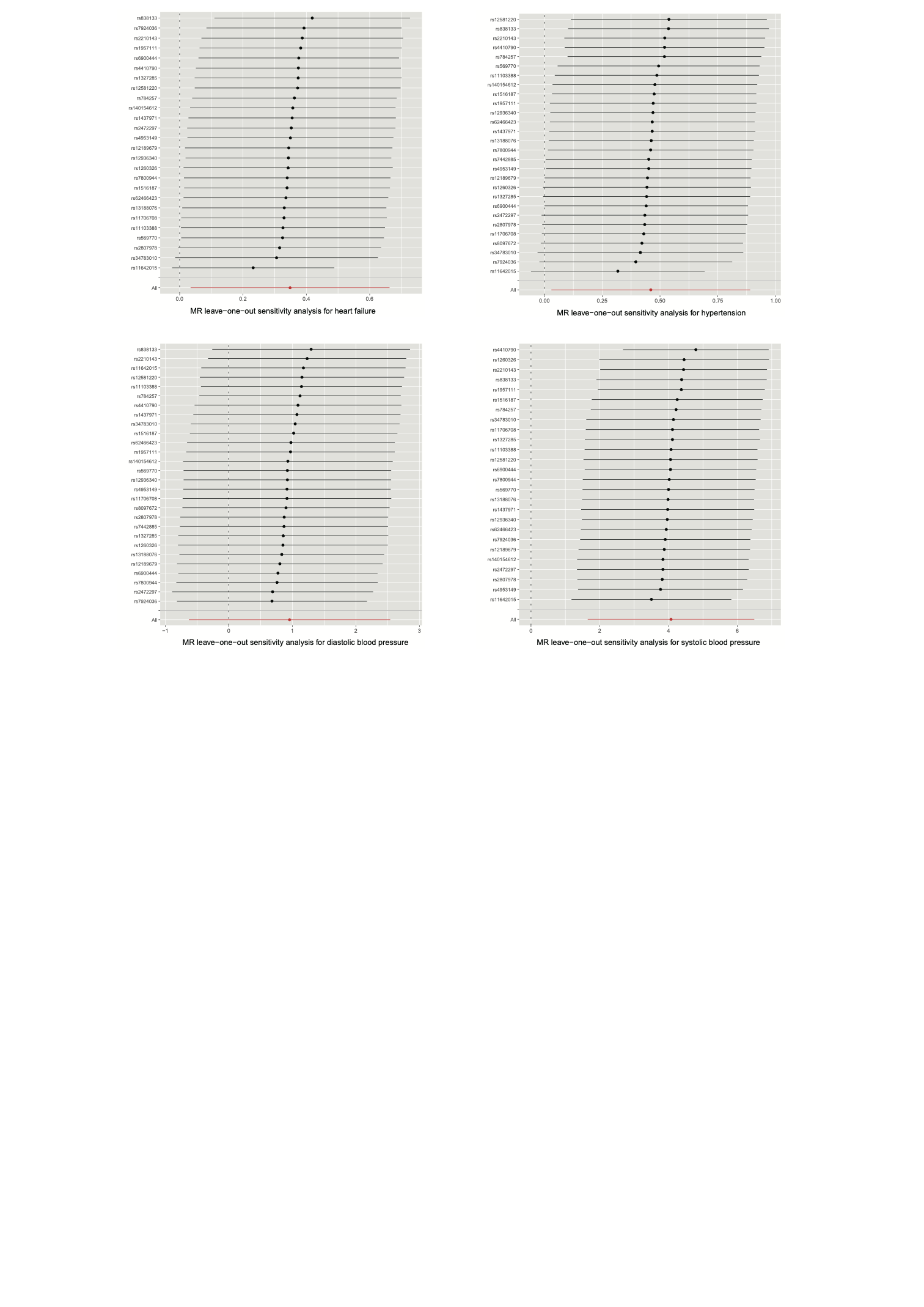

Supplement: Supplementary file 3 [file Image_3.TIFF]
