# Supplementary material for: Sodium intake and the risk of heart failure and hypertension: epidemiological and Mendelian randomization analysis
Source: Front Nutr. 2024 Jan 26;10:1263554. doi: 10.3389/fnut.2023.1263554 (PMC10853369; doi:10.3389/fnut.2023.1263554)
Supplement: Supplementary file 4 [file Table_1.docx]

Supplementary Table 1 summary of outcome GWAS

| phenontype | sample size | case | ethnicity | SNPs | year | consortium |
| --- | --- | --- | --- | --- | --- | --- |
| Heart failure | 977323 | 47309 | european | 7773021 | 2020 | HERMES Consortium |
| hypertension | 218754 | 55917 | european | 16380466 | 2021 | finngen |
| Systolic blood pressure | 97656 |  | european | 8029645 | 2022 | Within family GWAS consortium |
| Diastolic blood pressure | 757601 |  | european | 7160619 | 2018 | International Consortium of Blood Pressure |

GWAS: Genome Wide Association Study
