# Supplementary material for: Sodium intake and the risk of heart failure and hypertension: epidemiological and Mendelian randomization analysis
Source: Front Nutr. 2024 Jan 26;10:1263554. doi: 10.3389/fnut.2023.1263554 (PMC10853369; doi:10.3389/fnut.2023.1263554)
Supplement: Supplementary file 5 [file Table_2.docx]

| SNP | chr | pos | Effect allele | Other  allele | P val | eaf | se | beta | Sample size | r2 | F |
| --- | --- | --- | --- | --- | --- | --- | --- | --- | --- | --- | --- |
| rs1194277 | 1 | 72714331 | G | C | 6.5E-10 | 5.1E-01 | 2.4E-03 | 1.5E-02 | 3.3E+05 | 1.1E-04 | 3.5E+01 |
| rs1260326 | 2 | 27730940 | C | T | 6.7E-16 | 6.1E-01 | 2.4E-03 | -2.0E-02 | 3.3E+05 | 1.8E-04 | 5.9E+01 |
| rs1516187 | 2 | 51930164 | C | T | 2.1E-09 | 8.7E-01 | 3.5E-03 | 2.1E-02 | 3.3E+05 | 1.0E-04 | 3.3E+01 |
| rs1437971 | 2 | 100986964 | C | A | 1.9E-11 | 6.5E-01 | 2.5E-03 | -1.7E-02 | 3.3E+05 | 1.3E-04 | 4.2E+01 |
| rs4953149 | 2 | 45157307 | C | T | 3.9E-08 | 3.4E-01 | 2.5E-03 | 1.4E-02 | 3.3E+05 | 8.6E-05 | 2.8E+01 |
| rs11706708 | 3 | 35699022 | T | C | 1.3E-10 | 2.1E-01 | 2.9E-03 | 1.9E-02 | 3.3E+05 | 1.2E-04 | 3.8E+01 |
| rs7442885 | 5 | 87682877 | G | C | 2.5E-11 | 2.1E-01 | 2.9E-03 | -1.9E-02 | 3.3E+05 | 1.3E-04 | 4.1E+01 |
| rs13188076 | 5 | 148099993 | T | G | 1.4E-08 | 2.3E-01 | 2.8E-03 | -1.6E-02 | 3.3E+05 | 9.0E-05 | 3.0E+01 |
| rs12189679 | 6 | 98333409 | A | G | 2.2E-11 | 4.7E-01 | 2.4E-03 | -1.6E-02 | 3.3E+05 | 1.3E-04 | 4.1E+01 |
| rs6900444 | 6 | 31241182 | T | C | 2.0E-08 | 4.7E-01 | 2.4E-03 | 1.3E-02 | 3.3E+05 | 8.9E-05 | 2.9E+01 |
| rs1327285 | 6 | 51246947 | G | A | 1.0E-15 | 2.3E-01 | 2.8E-03 | -2.2E-02 | 3.3E+05 | 1.8E-04 | 5.9E+01 |
| rs4410790 | 7 | 17284577 | C | T | 6.5E-12 | 6.3E-01 | 2.5E-03 | -1.7E-02 | 3.3E+05 | 1.3E-04 | 4.3E+01 |
| rs7800944 | 7 | 73035857 | C | T | 4.3E-10 | 2.9E-01 | 2.6E-03 | -1.6E-02 | 3.3E+05 | 1.1E-04 | 3.6E+01 |
| rs62466423 | 7 | 132064270 | T | C | 1.5E-08 | 8.5E-02 | 4.2E-03 | -2.4E-02 | 3.3E+05 | 9.0E-05 | 2.9E+01 |
| rs140154612 | 8 | 143495760 | C | T | 2.4E-09 | 2.0E-02 | 9.5E-03 | -5.7E-02 | 3.3E+05 | 1.3E-04 | 4.1E+01 |
| rs11103388 | 9 | 139107925 | A | G | 2.8E-09 | 3.3E-01 | 2.5E-03 | 1.5E-02 | 3.3E+05 | 9.9E-05 | 3.2E+01 |
| rs2807978 | 10 | 22208630 | C | T | 5.4E-11 | 7.1E-01 | 2.6E-03 | -1.7E-02 | 3.3E+05 | 1.2E-04 | 4.0E+01 |
| rs7924036 | 10 | 65191645 | T | G | 3.5E-08 | 5.0E-01 | 2.4E-03 | -1.3E-02 | 3.3E+05 | 8.5E-05 | 2.8E+01 |
| rs2210143 | 11 | 31300793 | G | T | 2.6E-11 | 3.2E-01 | 2.5E-03 | -1.7E-02 | 3.3E+05 | 1.3E-04 | 4.1E+01 |
| rs569770 | 11 | 118584498 | C | T | 2.6E-08 | 6.4E-01 | 2.5E-03 | 1.4E-02 | 3.3E+05 | 8.7E-05 | 2.8E+01 |
| rs12581220 | 12 | 78773251 | T | C | 2.0E-12 | 3.2E-01 | 2.6E-03 | -1.8E-02 | 3.3E+05 | 1.4E-04 | 4.6E+01 |
| rs1957111 | 14 | 29781178 | T | C | 3.4E-10 | 5.2E-02 | 5.4E-03 | 3.4E-02 | 3.3E+05 | 1.1E-04 | 3.6E+01 |
| rs2472297 | 15 | 75027880 | T | C | 3.0E-14 | 2.7E-01 | 2.7E-03 | -2.0E-02 | 3.3E+05 | 1.6E-04 | 5.3E+01 |
| rs11642015 | 16 | 53802494 | T | C | 1.1E-13 | 4.0E-01 | 2.4E-03 | 1.8E-02 | 3.3E+05 | 1.5E-04 | 5.1E+01 |
| rs12936340 | 17 | 3492998 | A | G | 3.7E-08 | 6.4E-01 | 2.5E-03 | 1.4E-02 | 3.3E+05 | 8.5E-05 | 2.8E+01 |
| rs784257 | 18 | 53397199 | C | T | 4.7E-08 | 8.1E-01 | 3.0E-03 | 1.7E-02 | 3.3E+05 | 8.4E-05 | 2.8E+01 |
| rs8097672 | 18 | 1839601 | T | A | 2.8E-08 | 1.5E-01 | 3.4E-03 | 1.9E-02 | 3.3E+05 | 8.7E-05 | 2.8E+01 |
| rs34783010 | 19 | 46180414 | T | G | 3.8E-13 | 1.9E-01 | 3.0E-03 | -2.2E-02 | 3.3E+05 | 1.5E-04 | 4.8E+01 |
| rs838133 | 19 | 49259529 | G | A | 3.1E-16 | 5.5E-01 | 2.4E-03 | 2.0E-02 | 3.3E+05 | 2.0E-04 | 6.5E+01 |
| total F value | |  |  |  |  |  |  |  |  |  | 1.1E+03 |

Supplementary Table 2: SNPs associated with urinary sodium as instrumental variables
